# Population Densities, Vegetation Green-Up, and Plant Productivity: Impacts on Reproductive Success and Juvenile Body Mass in Reindeer

**DOI:** 10.1371/journal.pone.0056450

**Published:** 2013-02-22

**Authors:** Torkild Tveraa, Audun Stien, Bård-J. Bårdsen, Per Fauchald

**Affiliations:** NINA – Norwegian Institute for Nature Research, FRAM – High North Research Centre for Climate and the Environment, Tromsø, Norway; The Ohio State University, United States of America

## Abstract

Global warming is expected to cause earlier springs and increased primary productivity in the Arctic. These changes may improve food availability for Arctic herbivores, but may also have negative effects by generating a mismatch between the surge of high quality food in the spring and the timing of reproduction. We analyzed a 10 year dataset of satellite derived measures of vegetation green-up, population densities, calf body masses and female reproductive success in 19 reindeer (*Rangifer tarandus*) populations in Northern Norway. An early onset of spring and high peak plant productivity had positive effects on calf autumn body masses and female reproductive success. In addition, body masses and reproductive success were both negatively related to population density. The quantity of food available, as determined by the onset of vegetation green-up and plant productivity over the summer were the main drivers of body mass growth and reproductive success. We found no evidence for an effect of the speed of spring green-up. Nor did we detect a negative mismatch between early springs and subsequent recruitment. Effects of global warming on plant productivity and onset of spring is likely to positively affect sub-Arctic reindeer.

## Introduction

Over the next century global warming is expected to result in a longer growing season [1] and a 50% increase in the above ground biomass in Arctic tundra [2]. Consequently it has been proposed that earlier springs and longer growing seasons will benefit ungulates, and alleviate negative impacts of climate change caused by more difficult winters with compacted and crusted snow or icing [3]. Evidence for a positive impact of earlier springs comes from studies of chamois (*Rupicapra rupicapra*) in France [4], red deer (*Cervus elaphus*) in Norway [5], [6] and Fennoscandian reindeer (*Rangifer tarandus*) [7], [8]. In addition, it has been suggested that the rate at which new high quality forage emerges is critical for ungulates [4], [9], [10]. The hypothesised mechanism is that when fresh vegetation emerge gradually throughout the season, high quality food is available over a longer time span than if vegetation green-up occurs in just a short time [6], [10]. Fast vegetation green-up has been shown to negatively affect juvenile growth in both bighorn sheep (*Ovis canadensis*) and mountain goats (*Oreamnos americanus*), and cause a reduction in juvenile survival of Alpine ibex [10]. For caribou in Greenland earlier springs have been suggested to result in a lower reproductive success [11], [12]. They suggest that *Rangifer* (caribou/reindeer) might be unable to adjust their timing of reproduction to the earlier surge of high quality food. Therefore earlier springs may cause a mismatch between optimal forage conditions and the timing of reproduction [11,12, see 13 for similar results and conclusions in birds]. Accordingly, concerns have been raised regarding the future viability of *Rangifer* in Arctic and sub-Arctic tundra ecosystems [14], [15].

The contrasting effects of early onset of spring in *Rangifer* in Greenland and Fennoscandia highlights the need for a better understanding of how a warmer climate affects large Arctic herbivores through effects on spring onset, the rate of vegetation green-up and plant productivity [12]. It should be recognised that interactions among these factors might preclude a generally applicable interpretation. For instance, it is possible that the benefits of an early onset of vegetation green-up might be lost in years with rapid vegetation green-up [16]. Furthermore, if a rapid green-up is associated with a high peak biomass, a reduction in access to high quality food may become compensated for by an increase in the quantity of forage. So far, no studies of *Rangifer* have investigated the interaction among these factors.

In addition, density dependent effects may strongly affect ungulates [17], [18] but this factor is seldom accounted for in the studies of *Rangifer* referred to above [but see 8]. Furthermore, it has been proposed that the effect of environmental conditions in early summer depends on population density, but so far, direct evidence supporting this is lacking [10]. In sum, both various aspects of spring phenology and density should be accounted for in further studies on this issue.

Reindeer in northern Norway have a higher reproductive success and are heavier in summers when overall plant productivity is high [19]. Here we expand on these findings using a large scale dataset of 19 reindeer populations with well-defined summer habitats and known densities. Using MODIS based vegetation monitoring data [20] we estimated the onset of spring, the rate of vegetation green-up and the peak plant productivity for each population. This allowed us to disentangle the importance of density, an early onset of vegetation green-up, the rate of vegetation emergence and peak plant productivity on autumn body masses of calves and the females’ reproductive success. The aim of the present study was to tease apart how various aspects of vegetation green-up affected body mass of calves and reproductive success of females, and how these relationships were affected by varying levels of density dependent food-limitation.

## Results

**Figure 1:**
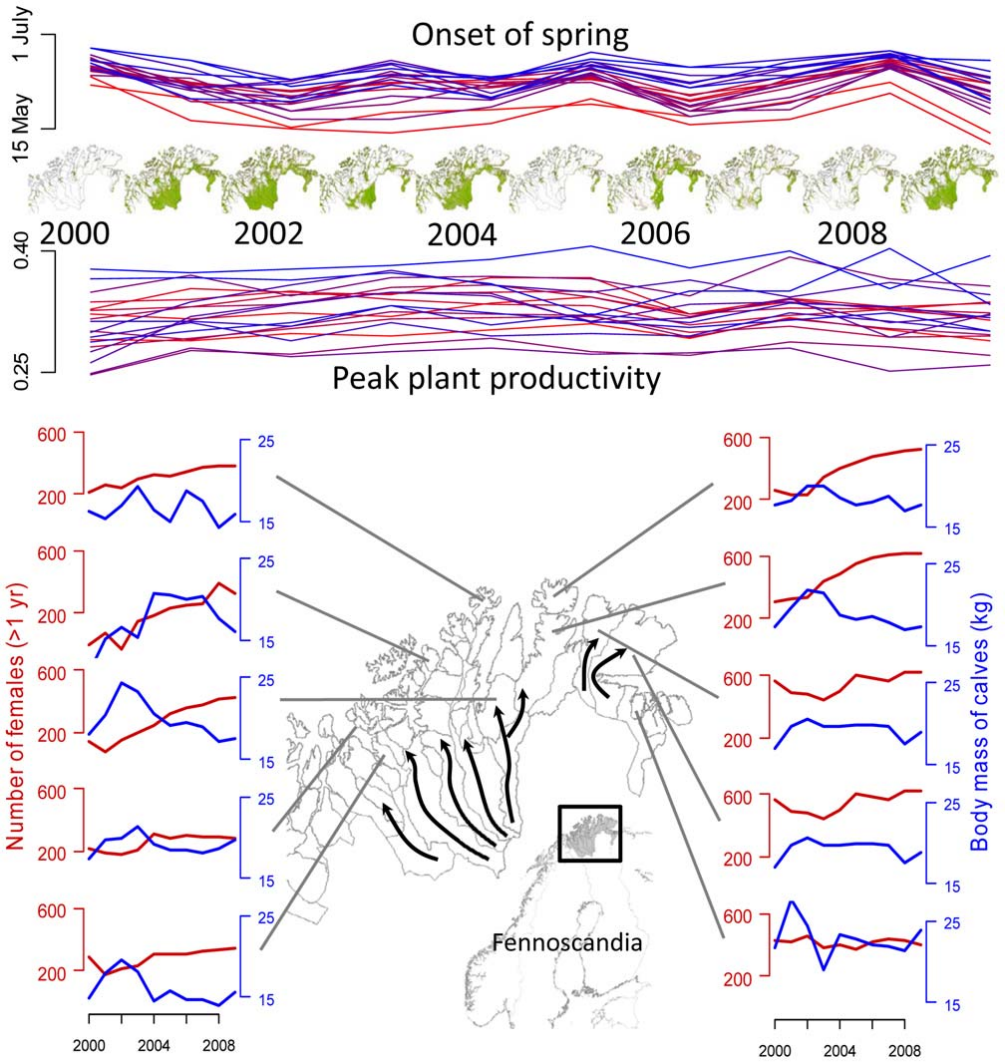
Top panel: Lines showing onset of spring and plant productivity for each population and MODIS 16 **day EVI composites from mid-May** (**days 129–144**)**.** Green areas have photo synthetic active vegetation. White areas are covered in snow/has no photo synthetic active vegetation. Main map: the extent of each reindeer herding district/population. Arrows in map indicate major migration routes between winter pastures in the interior and summer pastures along the coast. Time series (left and right panel) gives average number of females per reindeer herding unit (red lines) and body mass of slaughtered calves (blue lines) for 10 of the 19 populations included in the analysis.

The estimated range in onset of spring varied between 16–34 days within the study-populations over the 10 years of investigation. Overall, the latest onset of spring in a study population was estimated to the 24^th^ July (2000) and the earliest onset of spring was estimated to the 7^th^ May (2009). There was a high cross correlation between populations in onset of spring (mean correlation across study populations: r = 0.73, 95% CL  =  [0.71, 0.76], Figure 1), and even simple maps of EVI show that almost no green vegetation was available in mid-May in 2000, 2005 and 2008, while vegetation greening had come much further in mid-May in 2001, 2002, 2004 and 2006 (Figure 1). The between year variation in peak plant productivity was less synchronized across populations than onset of spring (mean correlation across study populations r = 0.30, 95% CL  =  [0.27, 0.34].

**Figure 2:**
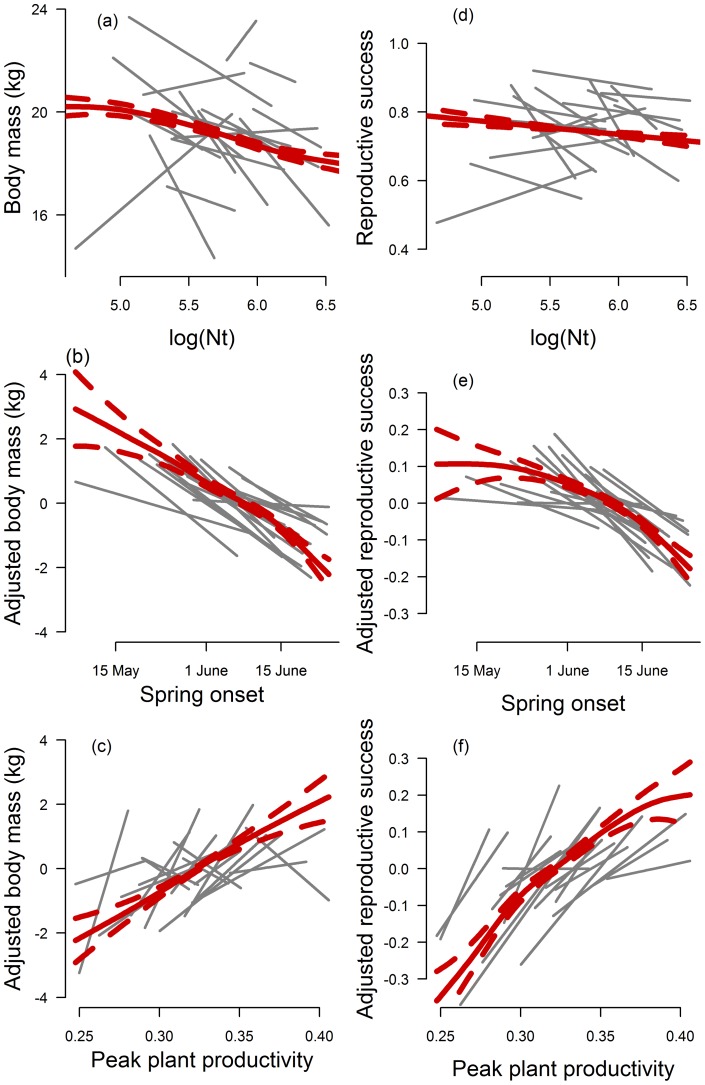
*Left panel* **:** (**a**) **The effect of number of females** (**log**[**Nt**])**,** (**b**) **onset of spring, and** (**c**) **peak plant productivity on body mass of calves slaughtered in the autumn.** *Right panel*: (d) The effect of number of females (log[Nt]), (e) onset of spring and (f) peak plant productivity on reproductive success. Red solid lines give estimated mean and standard error (red dashed lines) using generalized additive mixed modelling. Grey lines are estimated relationships for individual populations based on linear regressions.

The most parsimonious model describing body masses of calves in the autumn and reproductive success of females included population density, onset of spring and peak plant productivity (Supporting Information Table S1). The body mass of calves was on average higher in years with low population densities, an early onset of spring and high peak plant productivity (Table 1, Figure 2). Similarly, low population densities and an early onset of spring and high peak plant productivity also had a positive effect on female reproductive success (Table 1, Figure 2).

**Table 1:** Estimates from linear mixed-effects models (LME) for the model with lowest AIC relating calf body mass in autumn and reproductive success to population density (log[Number of females]), spring onset (SO) and peak plant productivity.

	Body mass		Reproductive success	
	β	95% CL	β	95% CL
Intercept	19.0	18.4, 19.6	0.74	0.696, 0.782
Population density	−1.22	−1.44, −1.00	−0.05	−0.068, −0.031
Spring onset (SO)	−0.10	−0.12, −0.09	−0.008	−0.009, −0.007
Peak plant productivity	26.3	20.3, 32.2	3.76	3.279, 4.234
*Random effects*:				
Among populations std	1.22	0.875, 1.72	0.089	0.062, 0.128
Among herding units std	0.72	0.55, 0.93	0.060	0.048, 0.075
Within herding units (residuals)	1.65	1.58, 1.73	0.139	0.134, 0.146

**Figure 3:**
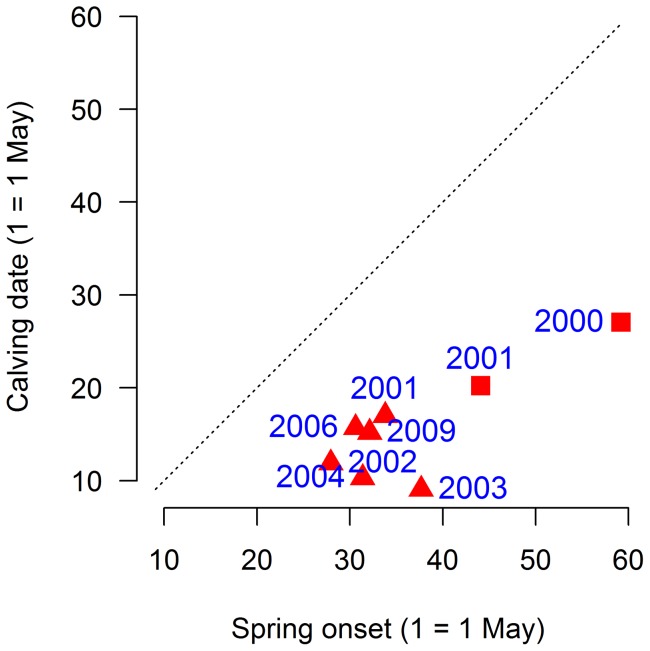
The relationship between calving date and spring onset for two herds with individually marked females. Squares are data from Spalca [37] and triangles are data from Njeaiddan [39]. The dashed line indicates the 1∶1 relationship.

Calving occurred two to four weeks prior to spring onset (mean  = 31.3, 95% CL  =  [16.0, 26.6], Figure 3). Calving was positively correlated to onset of spring only when data from both populations were included (r = 0.82, n = 7, p<0.05). When omitting two observations from the population with fewest number of observations, this relationship became negative and insignificant (r = −0.24, n = 5, p = 0.65). Body masses of calves in the previous fall, however, was negatively related to calving date both when data from both populations was used (r = −0.87, n = 7, p<0.01) as well as when the same two observations as above was excluded (r = −0.79, n = 5, p = 0.06) suggesting that previous fall calf body masses (index of herd body condition) was the most consistent predictor of calving dates.

## Discussion

We found that an early onset of spring and high peak plant productivity positively affected both reproductive success of females and autumn body masses of calves. Global climate change is expected to cause a shift towards earlier springs and an increase in plant productivity [1], [2], [21]. Reindeer population in our study region are therefore expected to experience improved reproductive success as a result of climate change. However, the density dependent effects observed in the study system suggest that population growth will counteract this positive effect of climate change.

Early onset of vegetation green-up and peak plant productivity had positive impact on body masses of calves in the autumn and the reproductive success of females in our study system. In contrast, the rate of vegetation green-up had no statistically significant impact on either female reproductive success or autumn calf body mass. This suggests that variation in quantity of forage is more critical than variation in quality of forage in this study system. In Arctic and sub-Arctic tundra ecosystems the growing season is short and primary productivity low compared to ecosystems at lower latitudes. This may explain the importance of food quantity as a limiting factor for reindeer in tundra ecosystems [22].

The rate of vegetation green-up has been interpreted as a measure of the temporal availability of high quality forage, as gradual emergence of new vegetation gives access to high quality, easily digestible, food over a long period [9], [10]. However, often satellite derived indices of vegetation growth are correlated meaning that disentangling the relative impact of various measures are difficult [4], [23], [24]. At least for two of the populations studied by Pettorelli and co-workers a rapid increase in vegetation green-up was associated with late vegetation green-up [cf. 16] implying that disentangling the relative importance of early onset of vegetation green-up and rate of increase in green-up would be difficult. We used only predictors with no pattern of colinearity, and we specifically tested the significance of each vegetation index on reindeer performance. Thus, we feel confident that colinearity did not influence our general conclusion and results.

Among our populations both positive and negative correlations between onset of spring and peak plant productivity were evident and both these vegetation indices affected gain in body mass of calves and reproductive success of females. This highlights the potential significance of incorporating more than just one measure of vegetation green-up, and ideally also several populations, when studying the impact of vegetation green-up on ungulates’ performance. In our case the onset of vegetation green-up was synchronous over a large spatial scale, while peak plant productivity was not (Figure 1). Such complex interactions have the potential to preclude the interpretation of results if positive effects of early vegetation green-up are counteracted by low plant productivity. They may also explain the contrasting effects of early vegetation green-up on *Rangifer* in Greenland and Fennoscandia [7], [8], [11]. It is perceived that early onset of springs are related to slow vegetation green-up [16], but this might not always be the case. Early onset of springs may, at least some times, be counteracted by rapid vegetation green-up, so that the positive effect of early vegetation green-up is lost. Thus, the underlying causes of different responses among populations should be further explored to understand the apparent differences in responses to climatic variation. Studies of red deer suggest that complex interactions between vegetation green-up in summer, and temperature and precipitation in winter play a key role in their dynamics [6]. The same processes are at work for reindeer. Deep snow hinder their access to food in winter, and the gradual snow melt at increasing elevations ensure that high quality forage is available for an extended period during summer.

As expected we found negative relationships between population size and body mass of calves and reproductive success of females. Contrary to our expectation [25], [26], however, we found no strong evidence in support of the hypothesis that variation in vegetation green-up has a stronger effect on the reindeer in years with high population densities. Thus, our results are consistent with the results of Pettorelli and co-workers [10]. Pettorelli and co-workers [10] proposed that too little variation in population densities made it difficult for them to detect interactions in their study. In our study population sizes varied substantially over the study period (Figure 1). What should be noted in this context is that the reindeer populations in the study region are all kept at fairly high densities through predator control and low harvest rates. We cannot therefore exclude the possibility that the interaction effect would have been stronger if the variability in population densities included substantially lower densities [cf. 24].

According to the available data, calving in our region occurs in mid-May, two to four weeks prior to the onset of spring (Figure 3). This is in contrast to caribou at Greenland where calving takes place in the first half of June, or one to three weeks after onset of spring [12]. Although, the estimates of spring onset are not directly comparable due to methodological differences, the results suggest that reindeer in Fennoscandia might be in a better position with respect to climate change than the caribou in Greenland since advancing spring onset will reduce the mismatch between spring onset and calving in Fennoscandia while it will increase the mismatch in Greenland. The ability of reindeer to adjust the timing of birth to climate change may become crucial in the future. Our study suggests that the ability to advance birth is linked to body mass, which for reindeer is linked to density and climatic conditions in spring and summer (Figure 2). Earlier springs is expected to result in increased gain in mass during summer and advancement in date of birth, while increasing reindeer densities is expected to decrease body masses and delay calving (see also [27]). Management actions that regulate reindeer densities might thus become crucial to ensure that calving in reindeer match vegetation green-up.

Throughout their circumpolar range reindeer have shown contrasting patterns in population trends over the last few decades [14], [24], [28]. However, with as many as 80% currently declining concern has been raised regarding their future in Tundra ecosystems under the expected climate change [14]. Climate change is predicted to cause both warmer and wetter winters and an earlier spring green-up [1]. A number of studies correlating population trends to large scale weather phenomena have documented that reindeer [24], [28]–[30] as well as other herbivores suffer in warm and wet winter climate [31]–[34]. The observed spatial variability in the population trends of *Rangifer* populations may therefore be due to spatial variation in how warmer and wetter winters affect winter food availability, and how warmer summers affect food availability. In this context, we note that growth rates of Svalbard reindeer (*Rangifer tarandus platyrhyncus*) populations living in an oceanic environment were negatively affected to the amount of snow falling throughout the winter, while a population living in a more continental environment at Svalbard was not [35]. Contrasting effects of various climatic variables have also been found for Norwegian moose *Alces alces* populations covering a substantial gradient in climate [36]. The relative change in winter climate, with associated effects on winter food availability, versus the change in vegetation spring green-up and its consequences for summer food availability, appear to be key factors in forecasting the future of *Rangifer* in tundra ecosystems [34].

## Materials and Methods

### Study System

Semi-domestic reindeer in Norway have shown the same response to climatic conditions as has been reported for wild reindeer and caribou. Notably, the northern populations fluctuate in concert with the climatic conditions in the winter. The populations collapsed at the beginning of this century following a historic population peak in the late 1980s/early 1990s and a series of difficult winters in the late nineties [24].

Reindeer densities are generally high due to extensive predator control and low harvest rates in Northern Norway [37]. As in most other Arctic regions, reindeer herds in Northern Norway undertake long-distance migrations between winter pastures in the interior and summer pastures along the coast (Figure 1). An extensive network of fences in combination with natural barriers (notably coastlines and mountain ranges) ensures that the various populations are kept within their designated area. Animals are free ranging year round and gathered a few times a year for population counts, calf marking and slaughtering. The total number of animals for each sex and age (calves or older) is counted by the herders annually in late winter, and reproductive success is measured as the number of calves counted in summer or early autumn prior to the rut (mid-September) divided by the number of adult females in the previous winter. Each calf is captured and earmarked according to owner. Slaughtering takes place from September – March, with the most intensive slaughtering period being in September, i.e. after calf marking and prior to the rut. Herders have to report data regarding population size, recruitment and losses to the Reindeer Husbandry Administration annually. Control counts of each herder’s population size are undertaken by the authorities roughly every second year. An extensive subsidy system ensures that most animals (>90%) are slaughtered at government approved slaughter houses that report these data to the Reindeer husbandry administration. Sex ratio is highly skewed towards females in these systems with an average of 10% of the adults in the herds being males (range 4–27%). We restricted our study to 19 management districts that slaughtered calves annually over the period 2000–2009. We focus on autumn calf body mass, as obtained from slaughter houses, as a measure of herd condition in the autumn. Many calves but few adult females are slaughtered annually, giving much better annual estimates of calf body masses than adult female body masses. Previous studies have demonstrated that calf body masses are more strongly influenced by changes in population densities and climate than female body masses [19], [38], [39]. Accordingly, Morellet and co-workers [40] proposed that the body mass of calves is a good indicator of food availability in ungulates. Altogether 155,781 calves were slaughtered in 2000–2009 between 1 September and 31 March. We restricted our analysis to data from 127,019 calves slaughtered between 1 September and 31 December to remove possible effects of late winter feeding conditions on calf body masses. (see Supporting Information Table S2 for overview of body mass data in each population).

The vegetation in the region is dwarf-shrub dominated of a low-Arctic/low-alpine type [41]. Narrow belts of mountain birch (*Betula pubescens*) extends up to 300–500 m a.s.l. in the relatively benign south-west section of the study area, whereas the forest border is set at 0–100 m a.s.l. in the colder north east [42]. Details about the study system can be found in [40], [43].

To examine when calving occurred relative to onset of spring, we compiled data for 2000–2001 [see 37] and for 2001–2004, 2006 and 2009 [see 39] from two herds (Spalaca and Njeiaddan, respectively) where we had access to data on the calving dates of individually marked females.

### Vegetation Indices and Vegetation Green-Up

We used high quality remote sensing data from the MODIS platform [20], [44] to determine the spring onset on the calving ground of the 19 populations of free-ranging animals covering c. 65 000 km^2^. The data is collected by NASA and available at (http://modis-land.gsfc.nasa.gov/vi.html). We used the 250 m 16 d Enhanced Vegetation Index (EVI) composite version 5 from the Terra platform which is available since the end of 1999. NDVI and EVI are highly correlated in the area [19]. However, following the recommendation of Heute and co-workers [20], we used EVI rather than NDVI because EVI is less sensitive to temporal variation in canopy background which is particularly relevant in open canopies such as the tundra. The equation for EVI takes the form
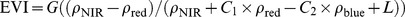
(1)Where *ρ* are atmospherically corrected surface reflectance, *L* is the canopy background adjustment that addresses nonlinear, differential NIR (near infra red) and red radiant transfer through a canopy, and *C*_1_, *C*_2_ are the coefficients of the aerosol resistance term, which uses the blue band to correct for aerosol influences in the red band. The coefficients adopted in the EVI algorithm are, *L* = 1, *C*_1_ = 6, *C*_2_ = 7.5, and *G* (gain factor)  = 2.5 [20]. The Terra platform has daily bypasses and due to sensor orbit overlap up to 64 observations per pixel is potentially available for each 16 day period. The 16 d composites are based on the best available data for each pixel as judged from viewing angle, presence of clouds and atmospheric contamination [20]. In addition, a quality mask marks the quality of each pixel so that pixels covered with e.g. clouds throughout the 16 d period are easily masked out. The MODIS product also includes a raster giving the acquisition date for each pixel. This information was used in our calculation of the timing of the annual development of phenology.

Prior to calculation of vegetation phenology we removed pixels containing lakes, and glaciers based on detailed maps of Norway. Additionally, we removed pixels consisting of bare rocks by comparing EVI maps with ground truth data, i.e. where maximum EVI <0.15 and coefficient of variation (cv) in EVI >1 based on all 16 day composites for the period 2000–2009. Pixels covered in clouds were also removed from the analysis based on the image reliability raster. Pixels classified as containing snow were however not removed as their value is ∼0 and as snowmelt is an important determinant of onset of spring. Beck and co-workers [45] recently proposed that a double logistic function was appropriate for describing vegetation dynamics at high latitudes based on MODIS time series data of vegetation. This method captures well the timing of bud-burst in the study region [46] and among-year variation in vegetation phenology [47]. For each pixel, we fitted the double logistic function proposed in [45] to the annual time series of EVI, but rather than imputing minimum EVI observed in October-November for the dark season, when no images were available, we imputed EVI = 0 because this more accurately describes the transition from snow on ground to green vegetation. The development of EVI in a given pixel and year with respect to time (*t*) was accordingly defined by:
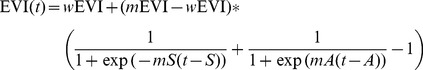
(2)where *w*EVI and *m*EVI is the estimated minimum and maximum EVI during the year, respectively, *S* is the date when predicted EVI  =  (*m*EVI−*w*EVI)/2 in the spring, *A* is the date when predicted EVI  =  (*m*EVI−*w*EVI)/2 in the autumn and *mS* and *mA* are the rates of EVI increase and decrease, respectively, in spring and autumn. Also contrary to [45], we did not refit our model using weights depending on the observations residual value in the fit. The reason for omitting this step was that initial analyses demonstrated this had no practical impact on the estimation of *S*, and because omitting this step halved the time needed to compute estimates for these parameters. The annual average in the estimates of *S* (eq. 2) over all pixels used by a reindeer population was used as a year and population specific index of the onset of spring (see Supporting Information Table S3 for sample sizes and Supporting Information Figure S1 to identify the location of each population). Hereafter we refer to this measure as the onset of spring. Using the same procedure, we used the average *mS* as a measure of the rate of green-up in spring and the average *mEVI* as a measure of peak plant productivity [4], [48]. For comparison with an earlier study, we also calculated the summed EVI over all pixels from day 65–289 [19]. GIS work was done in GRASS [49] and the computation of development of EVI (eq. 2) was programmed using a constrained optimization procedure [50] implemented in the in the ‘scipy’ library in Python.

### Statistical Analyses

Since various measures of phenology using satellite derived measures are often strongly correlated [24], we examined the correlation between the different indices before using them as predictor variables.

There was no consistent temporal pattern of correlations between the onset of spring and rate of green-up within the 19 reindeer populations (r = 0.09, [−0.07, 0.24]) (mean Pearson’s r, [95% confidence limits, CL]) or between the onset of spring and peak plant productivity (maximum EVI) (r = 0.06, [−0.07, 0.20]). Earlier springs resulted in a higher summed EVI (a higher total season greenness) (r = −0.44, [−0.55, −0.33]). Rate of green-up was weakly negatively correlated to peak plant productivity (r = −0.19[−0.31, −0.07]). Peak plant productivity was, positively correlated to summed EVI (r = 0.54, [0.41, 0.67]). Based on these correlations, we continued our analyses using onset of spring, rate of green-up and peak plant productivity as independent predictor variables.

We developed predictive models in a linear mixed model framework using the library nlme [51] in R [52]. The data had a nested structure with reindeer herding unit nested in district (hereafter population), and we used this structure with random intercepts only. We modelled calf body mass in the autumn and reproductive success as a function of density of females per reindeer husbandry unit (log[*N_t]_*), onset of spring, rate of green-up, and peak plant productivity. To explore if the responses to vegetation green-up changed with increasing densities of females, we included the interaction between density of females and the various measures of spring phenology. Because the number of females per reindeer herding unit was counted in the winter and the number of calves was counted in the following summer, the number of calves sometimes exceeded the number of females, probably because some females were not seen in winter. For those reasons we could not model reproductive success using a binomial distribution. We considered modelling reproductive success using number of calves as the response variable, number females as the offset variable and a Poisson error distribution. However due to the large number of calves marked in each population per year (mean  = 1981.1) the assumtion of a Gaussian distribution for both reproductive success (number of calves per female >1 yr) and calf body mass seems reasonable. Since the dataset was limited to ten years and we had eight potential predictor variables, we used a forward model selection procedure. Based on our a priori expectations, we kept population size in all analyses and examined the model fit including one of the bio-climatic measures (onset of spring, rate of green-up, peak plant productivity) at a time. We refitted the model and included the bio-climatic predictors that alone reduced the AIC and included interaction terms between our a priori predictors (density or body mass) and the climatic predictors, to test the hypothesis that the responses to climatic variation changed with changes in density [cf. 10]. As above, the inclusion of interaction terms was evaluated using AIC. Among the top ranked models, we chose the model with fewest parameters and ΔAIC <2 following the principle of parsimony [53].

To visualise the underlying data of the most parsimonious mixed model we plotted the predicted values from linear regression models for each population, using partial residuals plots for aggregated data, and overlayed the average predicted values using the plotting tools available for generalized additive mixed model in the mgcv library [54].

## Supporting Information

Figure S1 **Overview of study area with specification of summer grazing areas for each population.** Number in figure corresponds to those in Supporting Information Table S2.
(TIF)

Table S1 **Model selection for the analyses of** (**a**) **calf body mass in autumn and** (**b**) **female reproductive success.** The model with ΔAIC <2 are outlined in bold.
(DOCX)

Table S2 **Overview of number of reindeer herding units within in each population and average body mass** (**x**) **in kilograms and number of calves slaughtered, i.e. sample size** (***n***)**.**
(DOCX)

Table S3 **Overview of number pixels available for calculation of vegetation green-up in spring for each population and year.** MapID refer to the number in Supporting Information Figure S1.
(DOCX)
