# Supplementary figures and images for: Population Densities, Vegetation Green-Up, and Plant Productivity: Impacts on Reproductive Success and Juvenile Body Mass in Reindeer
Source: PLoS One. 2013 Feb 22;8(2):e56450. doi: 10.1371/journal.pone.0056450 (PMC3579868; doi:10.1371/journal.pone.0056450)

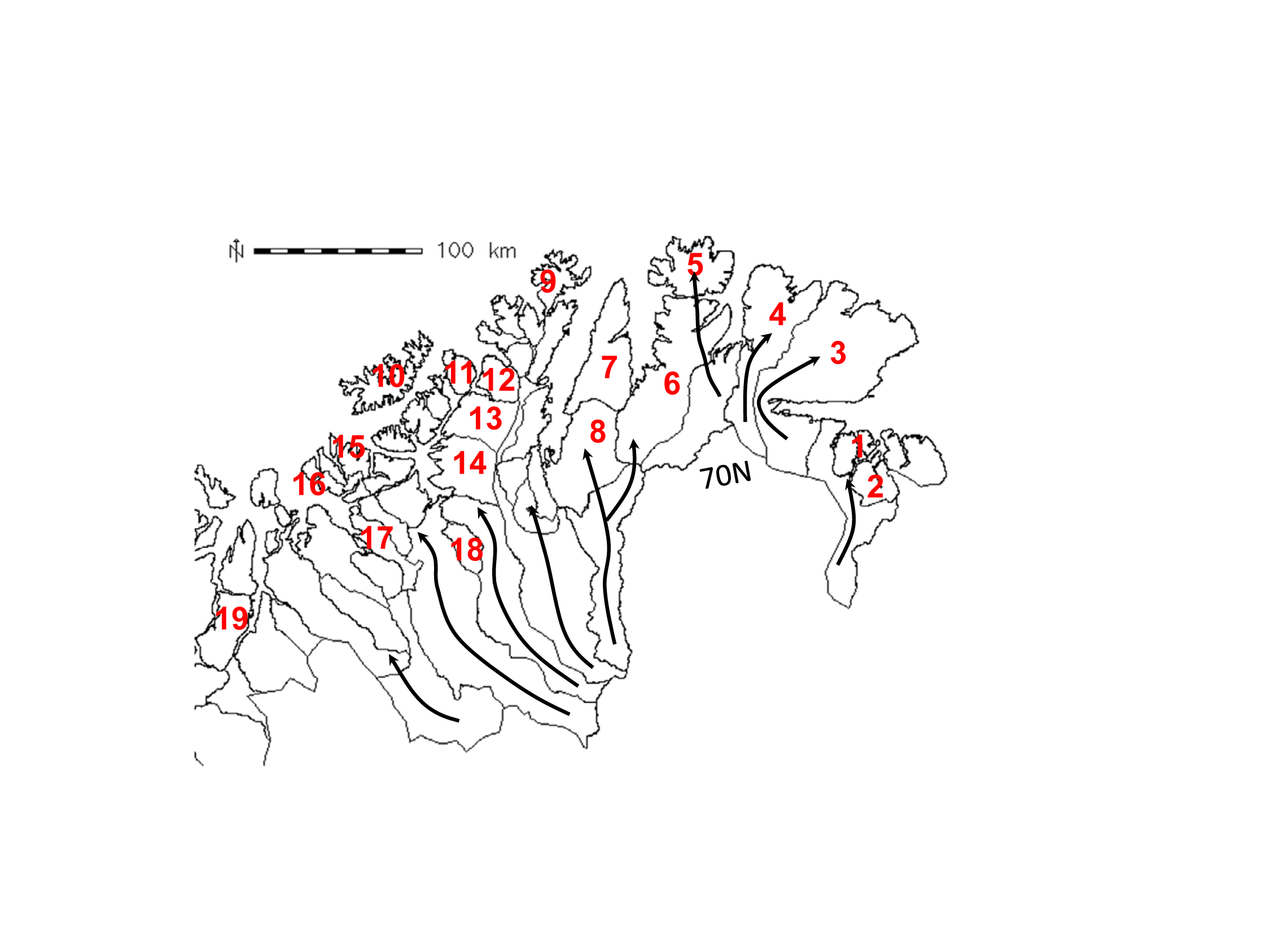

Supplement: Figure S1 — Overview of study area with specification of summer grazing areas for each population. Number in figure corresponds to those in Supporting Information Table S2. (TIF) [file pone.0056450.s001.tif]
