# Supplementary material for: Population Densities, Vegetation Green-Up, and Plant Productivity: Impacts on Reproductive Success and Juvenile Body Mass in Reindeer
Source: PLoS One. 2013 Feb 22;8(2):e56450. doi: 10.1371/journal.pone.0056450 (PMC3579868; doi:10.1371/journal.pone.0056450)
Supplement: Table S1 — Model selection for the analyses of (a) calf body mass in autumn and (b) female reproductive success. The model with ΔAIC <2 are outlined in bold. (DOCX) [file pone.0056450.s002.docx]

|  | | | | | | | | | | |
| --- | --- | --- | --- | --- | --- | --- | --- | --- | --- | --- |
|  | Population size (N_t_) | Spring onset (SO) | Rate of green-up  (RG) | Peak plant productivity (PP) | N_t_ *SO |  | N_t_ *PP | K | AIC | Delta AIC |
| (a) Body mass of calves: | | |  |  |  |  |  |  |  |  |
|  | X |  |  |  |  |  |  | 5 | 5738.3 | 259.3 |
|  | X | x |  |  |  |  |  | 6 | 5549.5 | 70.3 |
|  | X |  | x |  |  |  |  | 6 | 5740.4 | 261.3 |
|  | X |  |  | x |  |  |  | 6 | 5692.4 | 213.2 |
|  | **X** | **x** |  | **x** |  |  |  | **7** | **5479.4** | **0.21** |
|  | **X** | **x** |  | **x** | **x** |  | **x** | **9** | **5481.1** | **1.9** |
|  | X | x | x | x |  |  |  | 8 | 5481.3 | 2.2 |
|  | **X** | **x** |  | **x** | **x** |  |  | **8** | **5479.2** | **0** |
|  | X | x |  | x |  |  | x | 8 | 5481.4 | 2.2 |
| (b) Female reproductive success: | | | | | | | | | | |
|  |  |  |  |  |  |  |  |  |  |  |
|  | X |  |  |  |  |  |  | 5 | -952.1 | 351.0 |
|  | X | x |  |  |  |  |  | 6 | -1086.7 | 216.4 |
|  | X |  | x |  |  |  |  | 6 | -951.4 | 351.7 |
|  | X |  |  | x |  |  |  | 6 | -1123.5 | 179.6 |
|  | **X** | **x** |  | **x** |  |  |  | **7** | **-1301.9** | **1.21** |
|  | **X** | **x** |  | **x** | **x** |  | **x** | **9** | **-1302.1** | **0.97** |
|  | **X** | **x** |  | **x** | **x** |  |  | **8** | **-1303.1** | **0** |
|  | X | x |  | x |  |  | x | 8 | -1300.8 | 2.3 |
|  |  |  |  |  |  |  |  |  |  |  |
