# Supplementary material for: Population Densities, Vegetation Green-Up, and Plant Productivity: Impacts on Reproductive Success and Juvenile Body Mass in Reindeer
Source: PLoS One. 2013 Feb 22;8(2):e56450. doi: 10.1371/journal.pone.0056450 (PMC3579868; doi:10.1371/journal.pone.0056450)
Supplement: Table S2 — Overview of number of reindeer herding units within in each population and average body mass (x) in kilograms and number of calves slaughtered, i.e. sample size (n). (DOCX) [file pone.0056450.s003.docx]

| **Population** | **Units** |  | **2000** | **2001** | **2002** | **2003** | **2004** | **2005** | **2006** | **2007** | **2008** | **2009** |
| --- | --- | --- | --- | --- | --- | --- | --- | --- | --- | --- | --- | --- |
| Vestre-Sørvaranger | 4 | x | 19.2 | 20.8 | 20.3 | 19.4 | 18.6 | 17.7 | 18.5 | 19.1 | 16.5 | 20.5 |
|  |  | *n* | *2012* | *486* | *592* | *436* | *879* | *611* | *503* | *569* | *374* | *450* |
| Pasvik | 5 | x | 21.7 | 27.6 | 24.4 | 19.0 | 23.3 | 22.7 | 22.0 | 21.9 | 21.4 | 23.8 |
|  |  | *n* | *792* | *5* | *403* | *1* | *163* | *926* | *1198* | *25* | *22* | *775* |
| Várjjatnjárga | 15 | x | 17.0 | 19.7 | 20.6 | 19.7 | 19.7 | 19.9 | 19.9 | 19.8 | 17.5 | 18.9 |
|  |  | *n* | *71* | *834* | *1282* | *985* | *1225* | *2469* | *2316* | *2689* | *4533* | *2649* |
| Rákkonjárga | 6 | x | 21.1 | 19.7 | 21.9 | 21.4 | 21.2 | 21.8 | 22.5 | 22.3 | 20.1 | 22.8 |
|  |  | *n* | *26* | *367* | *588* | *938* | *1127* | *1258* | *1326* | *1239* | *487* | *1154* |
| Corgas | 10 | x | 17.6 | 18.1 | 19.9 | 20.0 | 18.6 | 17.7 | 18.0 | 18.6 | 16.9 | 17.6 |
|  |  | *n* | *60* | *269* | *493* | *702* | *1092* | *836* | *1316* | *1338* | *1875* | *2018* |
| Lágesduottar | 19 | x | 17.3 | 19.7 | 21.8 | 21.4 | 18.8 | 18.2 | 18.6 | 17.9 | 17.0 | 17.4 |
|  |  | *n* | *196* | *271* | *1070* | *907* | *1852* | *2553* | *2521* | *2862* | *2375* | *3160* |
| Spierttanjárga | 11 | x | 19.5 | 23.6 | 25.3 | 24.0 | 23.7 | 22.0 | 22.9 | 21.8 | 18.7 | 19.8 |
|  |  | *n* | *54* | *180* | *306* | *204* | *398* | *477* | *674* | *968* | *359* | *992* |
| Spierttagáisá | 22 | x | 18.0 | 20.3 | 24.4 | 23.3 | 20.6 | 19.1 | 19.6 | 19.0 | 17.1 | 17.5 |
|  |  | *n* | *19* | *83* | *443* | *596* | *940* | *702* | *1429* | *1847* | *1267* | *2025* |
| Máhkarávjju | 10 | x | 16.1 | 15.2 | 16.8 | 19.3 | 16.4 | 14.9 | 18.6 | 17.5 | 14.2 | 15.9 |
|  |  | *n* | *25* | *40* | *35* | *2* | *104* | *180* | *81* | *306* | *176* | *188* |
| Sállan | 10 | x | 18.2 | 20.0 | 22.9 | 22.8 | 20.8 | 19.5 | 21.7 | 21.9 | 21.6 | 21.4 |
|  |  | *n* | *65* | *69* | *51* | *156* | *223* | *258* | *419* | *426* | *163* | *871* |
| Fálá | 6 | x | 11.0 | 15.1 | 16.6 | 15.4 | 20.8 | 20.6 | 20.0 | 20.3 | 17.7 | 16.1 |
|  |  | *n* | *3* | *11* | *9* | *37* | *3* | *33* | *36* | *49* | *38* | *350* |
| Gearretnjárga | 8 | x | 18.9 | 18.8 | 22.5 | 20.7 | 19.6 | 18.4 | 19.7 | 19.0 | 18.2 | 18.8 |
|  |  | *n* | *65* | *69* | *51* | *156* | *223* | *258* | *419* | *426* | *163* | *871* |
| Fiettar | 14 | x | 17.6 | 19.7 | 21.0 | 20.1 | 17.1 | 16.5 | 18.2 | 18.5 | 16.4 | 16.7 |
|  |  | *n* | *183* | *363* | *545* | *833* | *1337* | *764* | *454* | *597* | *746* | *888* |
| Seainnus/Návggastat | 14 | x | 17.4 | 19.8 | 20.1 | 19.5 | 16.3 | 15.5 | 17.4 | 16.8 | 15.2 | 16.1 |
|  |  | *n* | *71* | *788* | *1522* | *1209* | *2904* | *1902* | *2336* | *3134* | *1694* | *1761* |
| Seakksnjárga | 3 | x | 20.3 | 20.2 | 20.1 | 20.6 | 18.7 | 18.5 | 17.4 | 17.4 | 17.0 | 19.0 |
|  |  | *n* | *50* | *81* | *90* | *77* | *125* | *127* | *163* | *228* | *202* | *252* |
| Silvvetnjárga | 6 | x | 17.5 | 19.6 | 19.9 | 21.3 | 19.1 | 18.4 | 18.4 | 18.0 | 18.7 | 19.7 |
|  |  | *n* | *119* | *143* | *248* | *62* | *294* | *280* | *330* | *346* | *354* | *498* |
| Spalca | 19 | x | 14.9 | 18.0 | 19.6 | 18.1 | 14.5 | 15.8 | 14.8 | 14.8 | 14.1 | 15.6 |
|  |  | *n* | *101* | *269* | *529* | *490* | *1231* | *548* | *1187* | *694* | *1016* | *634* |
| Beaskádas | 5 | x | 16.8 | 20.2 | 20.6 | 18.8 | 17.8 | 17.2 | 21.2 | 21.1 | 19.1 | 19.1 |
|  |  | *n* | *1* | *22* | *39* | *113* | *97* | *28* | *65* | *103* | *116* | *190* |
| Ivgoláhku | 3 | x | 18.5 | 20.9 | 22.2 | 19.0 | 17.1 | 17.8 | 19.1 | 18.6 | 19.2 | 18.7 |
|  |  | *n* | *9* | *18* | *54* | *84* | *326* | *170* | *259* | *397* | *306* | *401* |
