# Supplementary material for: Population Densities, Vegetation Green-Up, and Plant Productivity: Impacts on Reproductive Success and Juvenile Body Mass in Reindeer
Source: PLoS One. 2013 Feb 22;8(2):e56450. doi: 10.1371/journal.pone.0056450 (PMC3579868; doi:10.1371/journal.pone.0056450)
Supplement: Table S3 — Overview of number pixels available for calculation of vegetation green-up in spring for each population and year. MapID refer to the number in Supporting Information Figure S1. (DOCX) [file pone.0056450.s004.docx]

| **Name** | **MapID** | **2000** | **2001** | **2002** | **2003** | **2004** | **2005** | **2006** | **2007** | **2008** | **2009** |
| --- | --- | --- | --- | --- | --- | --- | --- | --- | --- | --- | --- |
| Vestre-Sørvaranger | 1 | 1820 | 2189 | 2044 | 1719 | 2187 | 2008 | 2028 | 2227 | 1880 | 1611 |
| Pasvik | 2 | 9789 | 9793 | 9789 | 9780 | 9790 | 9791 | 9783 | 9786 | 9782 | 9791 |
| Várjjatnjárga | 3 | 55591 | 57616 | 57752 | 58068 | 59741 | 60843 | 57115 | 58930 | 54462 | 53257 |
| Rákkonjárga | 4 | 15525 | 17244 | 16179 | 17353 | 18006 | 18484 | 17144 | 18727 | 15994 | 15760 |
| Corgas | 5 | 24351 | 25473 | 25537 | 26323 | 26849 | 27372 | 25831 | 26579 | 24885 | 23803 |
| Lágesduottar | 6 | 27778 | 31694 | 31209 | 31523 | 33133 | 34185 | 32719 | 33707 | 28670 | 27916 |
| Spierttanjárga | 7 | 16274 | 17755 | 17152 | 17763 | 18158 | 18060 | 17045 | 17956 | 16598 | 15697 |
| Spierttagáisá | 8 | 12197 | 16987 | 15020 | 15267 | 16315 | 17415 | 15655 | 17813 | 14839 | 13766 |
| Máhkarávjju | 9 | 4915 | 5394 | 4611 | 5233 | 5134 | 5427 | 5108 | 5531 | 5259 | 4455 |
| Sállan | 10 | 9276 | 9955 | 9261 | 9643 | 9663 | 9798 | 9873 | 9986 | 9559 | 9246 |
| Fálá | 11 | 2101 | 2655 | 2538 | 2435 | 2562 | 2797 | 2529 | 2515 | 2037 | 2116 |
| Gearretnjárga | 12 | 5578 | 5818 | 5775 | 5668 | 5951 | 5740 | 5675 | 5826 | 5553 | 5600 |
| Fiettar | 13 | 11281 | 11874 | 11617 | 11758 | 12030 | 11718 | 11746 | 12047 | 11652 | 11007 |
| Seainnus/Návggastat | 14 | 13888 | 15078 | 14647 | 14785 | 15020 | 14625 | 14898 | 14824 | 15195 | 14153 |
| Seakksnjárga ja Sildá | 15 | 1186 | 1293 | 1219 | 1254 | 1239 | 1237 | 1313 | 1425 | 1304 | 1218 |
| Silvvetnjárga | 16 | 2353 | 2388 | 2286 | 2394 | 2392 | 2384 | 2526 | 2663 | 2519 | 2313 |
| Spalca | 17 | 6427 | 7153 | 6956 | 7893 | 7675 | 7906 | 6398 | 8093 | 8107 | 5325 |
| Beaskádas | 18 | 4695 | 4853 | 4827 | 4768 | 4810 | 4574 | 4706 | 4825 | 4887 | 4523 |
| Ivgoláhku | 19 | 4546 | 4800 | 4731 | 4544 | 4841 | 4708 | 4777 | 4977 | 4634 | 4767 |
